# A hypothalamus‐lateral periaqueductal gray GABAergic neural projection facilitates arousal following sevoflurane anesthesia in mice

**DOI:** 10.1111/cns.70047

**Published:** 2024-09-24

**Authors:** Dan Wang, Chang Bao, Huimin Wu, Jiannan Li, Xinxin Zhang, Sa Wang, Fang Zhou, Huiming Li, Hailong Dong

**Affiliations:** ^1^ Department of Anesthesiology and Perioperative Medicine, Xijing Hospital The Fourth Military Medical University Xi'an Shaanxi China; ^2^ Key Laboratory of Anesthesiology (The Fourth Military Medical University) Ministry of Education of China Xi'an China

**Keywords:** arousal, GABAergic neuron, inhalation anesthetics, lateral hypothalamus, neurocircuits

## Abstract

**Background:**

The lateral hypothalamus (LHA) is an evolutionarily conserved structure that regulates basic functions of an organism, particularly wakefulness. To clarify the function of LHA^GABA^ neurons and their projections on regulating general anesthesia is crucial for understanding the excitatory and inhibitory effects of anesthetics on the brain. The aim of the present study is to investigate whether LHA^GABA^ neurons play either an inhibitory or a facilitatory role in sevoflurane‐induced anesthetic arousal regulation.

**Methods:**

We used fiber photometry and immunofluorescence staining to monitor changes in neuronal activity during sevoflurane anesthesia. Opto‐/chemogenetic modulations were employed to study the effect of neurocircuit modulations during the anesthesia. Anterograde tracing was used to identify a GABAergic projection from the LHA to a periaqueductal gray (PAG) subregion.

**Results:**

c‐Fos staining showed that LHA^GABA^ activity was inhibited by induction of sevoflurane anesthesia. Anterograde tracing revealed that LHA^GABA^ neurons project to multiple arousal‐associated brain areas, with the lateral periaqueductal gray (LPAG) being one of the dense projection areas. Optogenetic experiments showed that activation of LHA^GABA^ neurons and their downstream target LPAG reduced the burst suppression ratio (BSR) during continuous sevoflurane anesthesia. Chemogenetic experiments showed that activation of LHA^GABA^ and its projection to LPAG neurons prolonged the anesthetic induction time and promoted wakefulness.

**Conclusions:**

In summary, we show that an inhibitory projection from LHA^GABA^ to LPAG^GABA^ neurons promotes arousal from sevoflurane‐induced loss of consciousness, suggesting a complex control of wakefulness through intimate interactions between long‐range connections.

## Graphical abstract

In this study, we revealed an inhibitory projection from LHA^GABA^ to LPAG facilitates arousal from sevoflurane, suggesting a complex control of wakefulness through intimate interactions between long‐range connections. These findings enhance our understanding of the mechanisms of consciousness shift and potentially facilitate the development of studies involving the GABAergic system.

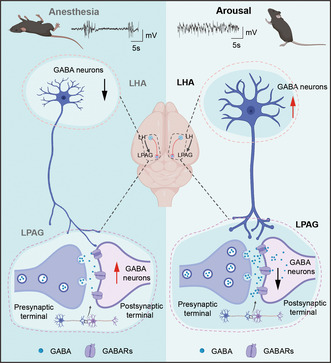

## 1 INTRODUCTION

Although considerable progress has been made in understanding the molecular mechanisms underlying the anesthetic effects of inhalational anesthetics, the process by which inhalational anesthetics such as sevoflurane induce loss of consciousness in neural circuits remains unclear.^1^, ^2^, ^3^ Inhibition of glutamate‐mediated excitatory transmission and facilitation of GABA‐mediated inhibitory transmission to produce anesthesia has long been accepted as the mechanism of general anesthesia.^4^, ^5^, ^6^ However, a number of recent studies have demonstrated that inhibitory neural pathways in specific brain regions are involved in facilitating emergence from general anesthesia.^7^, ^8^, ^9^ Conversely, GABAergic neurons in the nucleus accumbens (NAc) effectively facilitate the onset of the general anesthetic effect of propofol.^10^ Thus, these findings highlight the integral role of the inhibitory network in the process of reversible loss of consciousness induced by general anesthesia.^11^

The lateral hypothalamus (LHA) has been extensively studied for its contribution to the integration of neuronal responses with various peripheral signals associated with seeking and arousal.^12^, ^13^, ^14^, ^15^ Our previous research has shown that LHA hypocretin/orexin (Hcrt/Ox) neurons and their projections facilitate arousal from general anesthesia,^16^, ^17^, ^18^ while other work has demonstrated that activation of MCH neurons accelerates the REM state and reduces NREM sleep.^19^ Notably, LHA GABAergic neurons are also essential for regulating the sleep–wake transition. Optogenetic activation of LHA GABAergic neurons induces rapid wakefulness by directly modulating circuits within the thalamic reticular nucleus (TRN), locus coeruleus (LC),^20^ and dorsal raphe nucleus (DRN),^21^ with concomitant inhibition of the sleep‐promoting pathway controlled by the anterior hypothalamus.^22^ A previous study also showed that LHA^VGAT^ neurons inhibited the activation of orexinergic neurons in the local circuit during sleep.^23^ Therefore, it is necessary to further investigate the role of LHA^GABA^ neurons in the regulation of anesthesia‐arousal regulation of general anesthesia.

LHA^GABA^ neurons are reciprocally connected to several midbrain nuclei, including the periaqueductal gray (PAG) subregions.^13^, ^14^, ^24^, ^25^ The brainstem PAG has been implicated in the regulation of various pathophysiological functions, including motivation, pain perception, and threat responses, as the most important neuromodulator influencing sensation.^25^, ^26^, ^27^, ^28^ Recent studies have shown that the neuronal pathway LHA^GABA^ projections to the PAG modulate hunting and motivation.^13^, ^25^ Importantly, activation of LHA^GABA^‐VLPAG significantly controls REM sleep.^24^ Therefore, it is necessary to investigate whether and how LHA^GABA^ neurons and their downstream brainstem PAG modulate consciousness during general anesthesia.

To investigate the potential role of the LHA^GABA^‐LPAG neural projection during the sevoflurane anesthesia, in the current study we used a combination of in vitro immunostaining, in vivo fiber photometry, opto‐/chemogenetic stimulation and anterograde tracing techniques to investigate the function of LHA^GABA^ neurons in sevoflurane‐induced unconsciousness. Surprisingly, our results reveal how mutual disinhibition, mediated by long‐range inhibitory projections between its downstream brain area LPAG, facilitates arousal during sevoflurane anesthesia.

## 2 METHODS

### 2.1 Mice

All experimental protocols were approved by the Ethics Committee for Animal Experimentation and followed the Guidelines for Animal Experimentation of the Fourth Military Medical University (Xi'an, China) and ARRIVE (Animal Research: Reporting of In Vivo Experiments) guidelines. All experiments in this project were performed with adult C57BL/six male mice and transgenic male mice (8–15 weeks, 22–30 g), except for whole‐cell patch‐clamp experiments (>3 weeks old). C57BL/6J mice were obtained from Beijing Vital River Laboratory Animal Technology Co., Ltd., Vglut2‐IRES‐Cre and Vgat‐IRES‐Cre mice were originally provided by the Jackson Laboratory. All mice were kept in a specific pathogen‐free environment under a 12 h/12 h light/dark cycle (lights on from 7:00 a.m. to 7:00 p.m.) with ambient temperature (22–24°C), controlled humidity (38%–42%), and ad libitum access to food and water.

### 2.2 Experimental design

Activation of neurons in LHA and LPAG areas between O2‐2h group and Sevo‐2h group was observed by in vitro c‐Fos staining. The in vivo fiber photometry was recorded to study the activity changes of LHA^GABA^ neurons during the sevoflurane anesthesia and the process of arousal. Optogenetic combined chemogenetic modulation was performed to study the role of LHA^GABA^ neurons and their projections in LPAG during sevoflurane anesthesia‐induced unconsciousness, maintenance, and recovery. The downstream of LHA GABAergic neurons in the whole brain and the connection between the LHA GABAergic neurons and the LPAG were observed using anterograde tracing. To verify the electrophysiological characteristics in response to the activation of LHA GABAergic neurons during sevoflurane anesthesia, in vitro electrophysiology was used. Details are shown in the Figure S1 Flow Chart. Sample sizes were calculated via a Calculator from https://powerandsamplesize.com/Calculators/. Details can be seen in Material and Methods in Appendix S1 (Calculation of the sample size).

### 2.3 Optogenetic stimulation and EEG recording

To inhibit or activate LHA^GABA^ neurons, 2‐min yellow (580 nm, 1 Hz) or blue (473 nm, 20 Hz, 30 ms) laser pulses were used, respectively. Mice were habituated to the plexiglass container for 10 min. A heating mat was used to keep the mice warm during the whole stage of EEG recording. Electroencephalogram (EEG) signals were continuously recorded from mice under sevoflurane anesthesia using the PowerLab system and LabChart software.^29^, ^30^ A monitor measured sevoflurane levels in the container. The raw EEG data were bandpass‐filtered (0.3–50 Hz at a frequency of 1000 Hz) using a fourth‐order Butterworth filter. A threshold calculated from manually labeled suppression periods was applied to the transformed signals to obtain a binary series of burst and suppression states for each mouse. The EEG data were presented as a binary time series. The final step was to verify whether this time series corresponded to the original burst suppression waves. To determine the burst suppression ratio (BSR), we limited the minimum duration of the suppressed waves to 0.5 s, and the BSR was calculated as the percentage of time in each 1‐min window spent in the suppressed state. The absolute power spectrum in each time window was calculated using the spectrogram function provided by the MATLAB signal processing toolbox. The window length was 2 min pre‐stim and stim‐on during optogenetic stimulation. A window length of 4 s with an overlap of 3.9 s was used to set the parameters. Spectrogram is a time‐varying version of power spectrum estimated from successive windows of EEG data. The EEG signal was classified into 5 frequency bands as follows: delta (δ: 0.3–4 Hz), theta (θ: 4–10 Hz), alpha (α: 10–15 Hz), beta (β: 15–25 Hz), and gamma (γ: 25–50 Hz). As a percentage of the total power from 0.3 to 50 Hz, the relative power of each frequency band was calculated. In the experiments to observe the induction and emergence time, the optogenetic stimulation was applied continuously until the test mouse showed loss or recovery of consciousness behaviors, the cycle being 60 s on and 20 s off.

### 2.4 Measurement of induction and emergence time

For chemogenetic experiments, 3 mg kg^−1^ clozapine *N*‐oxide (CNO) or an equivalent volume of 0.9% saline was intraperitoneally injected into mice to manipulate LHA^GABA^ neurons. Behavioral tests were performed 30 min after the CNO injection. Anesthesia was induced and maintained using 2.4% sevoflurane and 1.5 L min^−1^ pure oxygen. The cylinder was rotated 90° every 15 s, and the induction time was measured as the interval from the start of anesthetic inhalation to loss of righting reflex (LoRR). Anesthesia was maintained for 30 min, and emergence time was defined as the interval from anesthesia cessation to return of righting reflex (RoRR). During anesthesia induction in optogenetic experiments, optical stimulation was administered at the start of sevoflurane inhalation and continued until LoRR. During emergence, the mice were optically stimulated from the end of sevoflurane inhalation until RoRR. A heating mat was used to keep the mice warm during the whole stage of measurement of induction and emergence. Meanwhile, the concentration of sevoflurane was measured by using Philips monitor (IntelliVue MX500).

### 2.5 Anterograde tracing and projection average intensity values

For anterograde tracing, 200 nL rAAV‐Ef1α‐DIO‐mCherry was microinjected into the LHA. The whole brain was sliced after 3 weeks of viral expression, and laser confocal fluorescence microscopy and ImageJ were used to count the projection intensity of LHA GABAergic neurons projection to each nucleus. Following the Allen Brain Atlas, the regions of interest (ROIs) and the boundaries for each regional area were plotted. The mean or average intensity was calculated for each region using ImageJ. The mean was calculated over all samples and averaged over four samples.

### 2.6 Statistical analyses

All analyses were conducted by investigators blinded to the experimental group. Statistical analyses were performed using Prism 9.0 (GraphPad Software, USA). Parametric data are presented as Mean ± SD. Data that met these conditions were analyzed using a two‐tailed unpaired, a one‐factor analysis of variance (ANOVA) and a two‐way (ANOVA) followed by Bonferroni correction. Except for the optogenetic experiments of the BSR assays, all behavioral, fiber photometry recording and cell counting data were collected by counterbalancing the experimental conditions with controls. Details of some of the statistical analyses carried out can be found in Table S2 (statistical table). Statistical significance was set at *p* < 0.05.

Further description of the methods (including Stereotaxic Surgery, Fiber Photometry Recording, Immunofluorescence Staining, Slice Recording and Calculation of the sample size) can be found in the Appendix S1. Details of key resources including mice, viruses, antibodies, software and instruments are shown in the Table S1.

## 3 RESULTS

### 3.1 Neural response of LHA^GABA^ neurons during sevoflurane anesthesia

**FIGURE 1:**
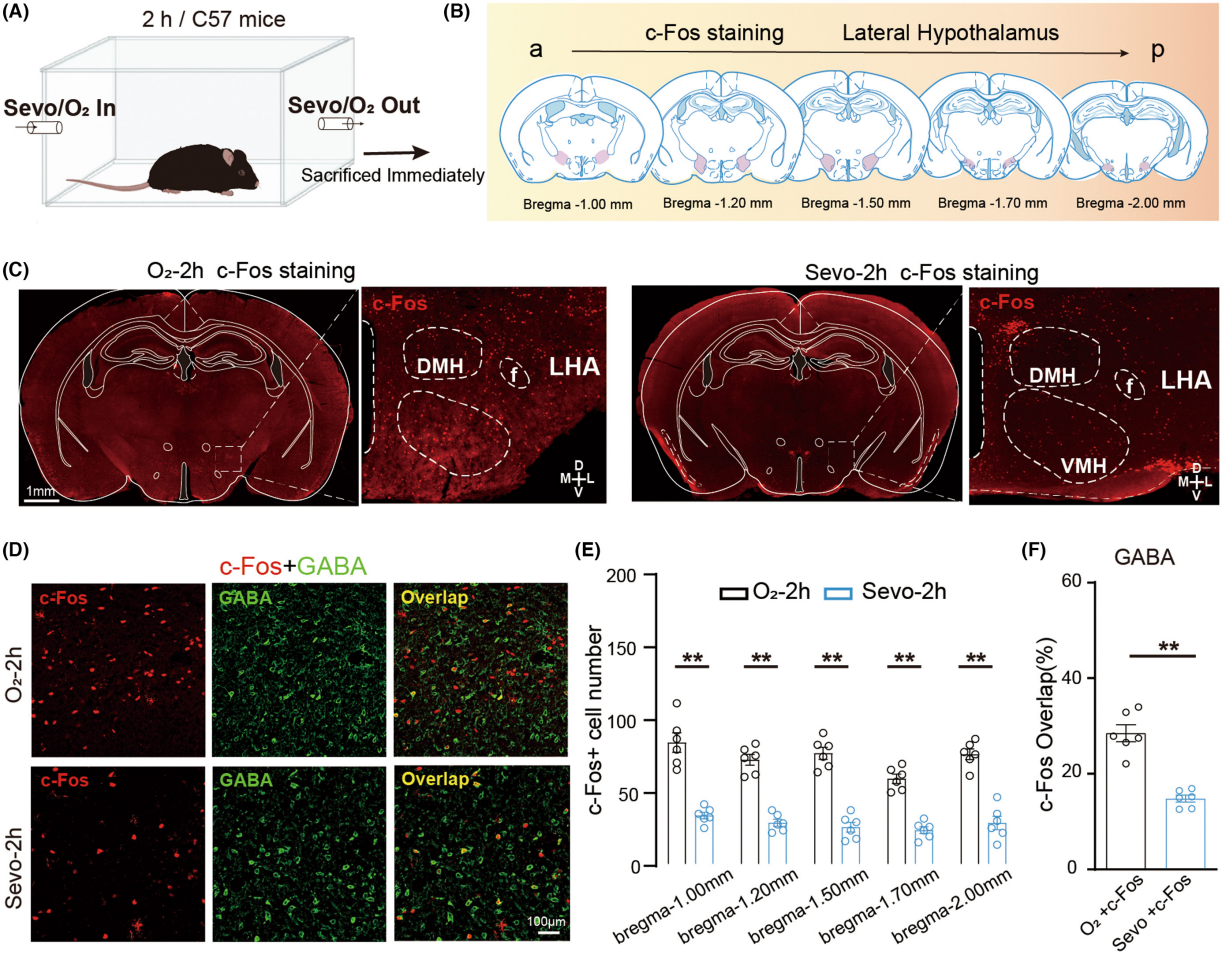
Sevoflurane anesthesia decreases the neural activity of LHA^GABA^ neurons. (A, B) Schematic representation of the brain slice experiment after inhalation of sevoflurane anesthesia or pure oxygen. (C) Representative images of c‐Fos expression in the brain regions of the control (O_2_‐2h) and sevoflurane anesthesia (Sevo‐2h) groups. (D) Representative micrographs showing overlapping GABA (green) and c‐Fos (red) stainings in the LHA after 2 h of oxygen exposure (O_2_‐2h) or sevoflurane anesthesia (Sevo‐2h). (E) Expression of c‐Fos in different planes of the LHA region from rostral to caudal. Two‐way ANOVA followed by post hoc Bonferroni's multiple comparisons: *F*[4,50] = 5.079, *p* = 0.0016. There are eight slices from four mice in each group. (F) Percentage of c‐Fos‐positive GABAergic neurons in the LHA of the control and sevoflurane groups. Two‐tailed unpaired Student's *t*‐test: *t*[10] = 4.464, *p* = 0.0012. There are eight slices from four mice in each group. *p < 0.05; **p < 0.01. Data are presented as Mean ± SD.

To investigate the neural activity of LHA neurons during sevoflurane anesthesia, we initially examined c‐Fos expression, an immediate early gene associated with neural activity. We anesthetized C57BL/6 male mice 2 h with 2.4% sevoflurane vaporized in oxygen at a flow rate of 1.5 L min^−1^ and sacrificed the mice immediately after that (Figure 1A,B). Immunofluorescence staining showed that c‐Fos expression decreased from rostral to caudal after 2 h of sevoflurane anesthesia (Figure 1C). Compared to the oxygen inhalation group, the percentages of c‐Fos‐positive neurons in the LHA were severely inhibited in the sevoflurane anesthesia group (Figure 1E). And, especially the interneuron of the LHA, GABAergic neurons were significantly lower as orexinergic and glutamatergic excitatory neurons in the sevoflurane anesthesia group (Figure 1F, Figure S2), indicating that these neurons were severely inhibited by sevoflurane anesthesia (Figure 1F, Figure S2D middle and right). This result revealed that LHA GABAergic neurons play its role in consciousness transition during anesthesia through other brain regions. We also analyzed the percentage of c‐Fos‐positive MCH neurons, suggesting that LHA^MCH^ neurons play a role opposite to other neurons (Figure S2A,D left). These results suggest that activity changes in LHA neurons, including LHA^GABA^ neurons, regulate arousal from sevoflurane‐induced anesthesia.

**FIGURE 2:**
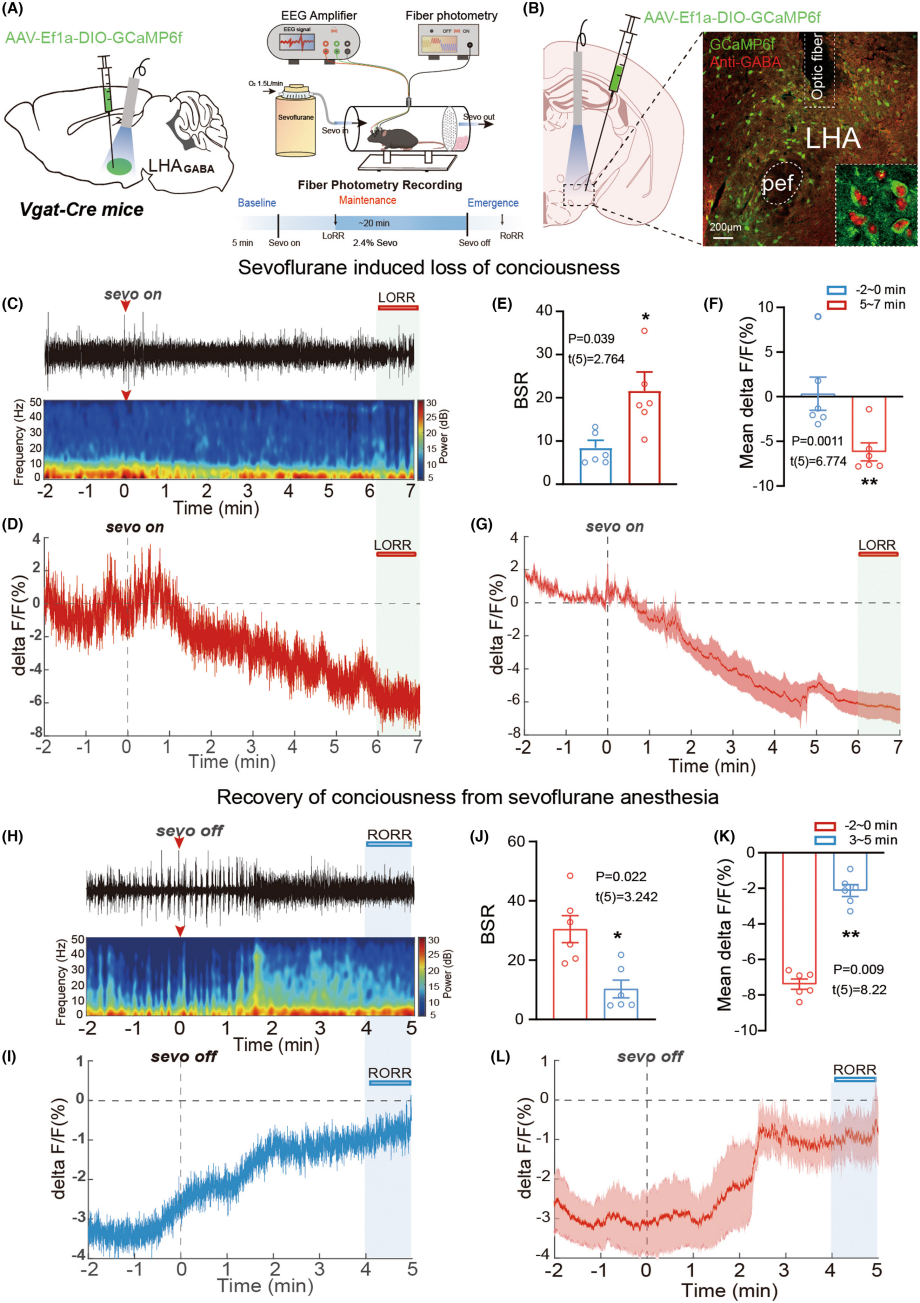
Calcium signals of LHA^GABA^ neurons responding to sevoflurane anesthesia. (A) Schematic illustration of calcium virus injection (left) and experimental equipment (right). (B) The GCaMP6f viral verification in the LHA slice. (C, D) Representative EEG and calcium recordings during loss of consciousness induced by sevoflurane anesthesia. (H, I) EEG and calcium levels were recorded during recovery of consciousness. Calcium signals decrease after the loss of consciousness (D) but increase after recovery of consciousness from sevoflurane anesthesia (I). (E, F, J, K) BSR and calcium activity changes during baseline, sevoflurane anesthesia, and emergence periods. Quantified EEG and GCaMP signals change between wake (before Sevo on, −2 min to 0 min) and LORR (Sevo on, 5 min to 7 min); the BSR of EEG recordings increases (E; *p* = 0.039, *t*[5] = 2.764), while GCaMP6f signals are significantly reduced (F; *p* = 0.001, *t*[5] = 6.774). Quantified EEG and GCaMP signals change between sevoflurane anesthesia (before Sevo off, −2 min to 0 min) and emergence (Sevo off, 3 min to 5 min); the BSR of EEG recordings decreases (J; *p* = 0.022, *t*[5] = 3.242), while GCaMP signals increase (K; *p* = 0.009, *t*[5] = 8.22). (G, L) Averaged delta F/F_0_ ratios of calcium signals from LHA^GABA^ neurons in response to sevoflurane‐induced LORR (G) and RORR (L). There are six mice in each group. *p < 0.05; **p < 0.01. Data are presented as Mean ± SD.

To further assess the in vivo activity of LHA^GABA^ neurons, we microinjected AAV‐DIO‐GCaMP6f into the LHA of Vgat‐Cre mice, achieving Cre‐dependent expression of calcium indicators in LHA^GABA^ neurons (Figure 2A,B) to observe alterations in neural activity in real‐time during anesthetic waking. After anesthesia initiation with sevoflurane, the calcium activity of LHA^GABA^ neurons gradually decreased (Figure 2D,F,G) while BSR notably increased (Figure 2C,E). After anesthesia termination, the calcium activity of mice recovered before they regained consciousness (Figure 2I,K,L), and BSR gradually decreased (Figure 2H,J).

These findings imply that LHA^GABA^ neurons were inhibited by sevoflurane anesthesia and reactivated during emergence from anesthesia, implying that LHA^GABA^ neurons regulate general anesthesia.

### 3.2 Manipulation of LHA^GABA^ neurons accelerates arousal and reduces BSR during sevoflurane anesthesia

**FIGURE 3:**
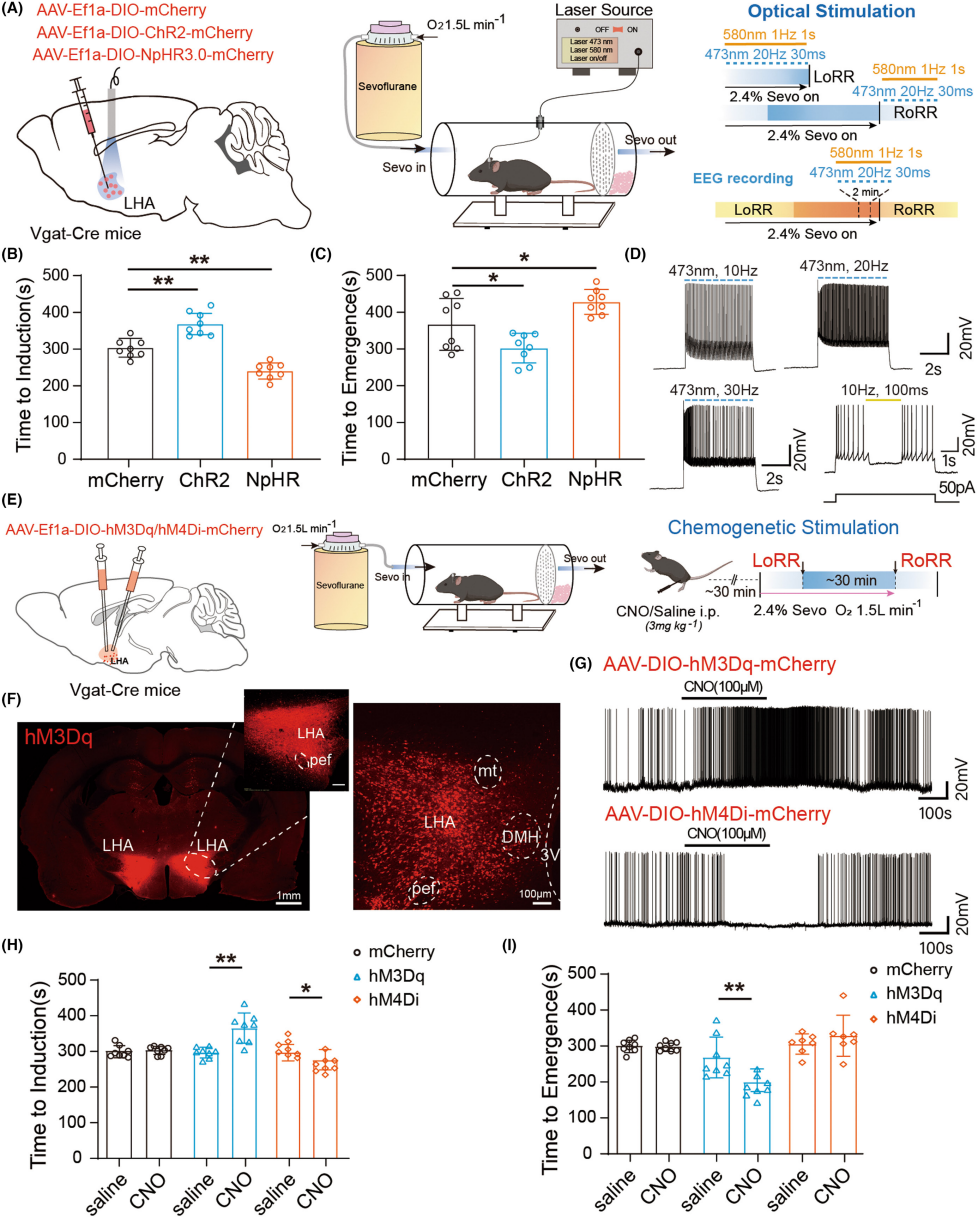
Optogenetic and chemogenetic manipulations of LHA^GABA^ neurons impact induction of and emergence from sevoflurane anesthesia. (A) Schematic illustrating the virus injection site (left) and the experimental paradigm for optogenetic manipulation combined with general anesthetic behavioral test (right). (B, C) Activation or inhibition of LHA^GABA^ neurons in response to anesthesia induction and emergence time. One‐way ANOVA followed by post hoc Bonferroni's multiple comparisons: LoRR, *F*[2,21] = 0.6234, *p* < 0.0001; RoRR, *F*[2,21] = 8.295, *p* = 0.0022. (D) Whole‐cell patch‐clamp recordings of LHA^GABA^ neurons activated in vitro in LHA slices by blue light of different frequencies. (E) Schematic illustration of chemogenetic viral conduction (left), experimental paradigm for chemogenetic stimulation, and general anesthetic behavioral test (right). (F) Verification of hM3Dq expression efficiency. (G) Representative traces of spontaneous firing upon CNO application to LHA^GABA^ neurons transfected with hM3Dq in LHA slices. (H, I) Effects of activation or inhibition of LHA^GABA^ neurons on induction of and arousal from sevoflurane anesthesia. Two‐way ANOVA followed by post hoc Bonferroni's multiple comparisons: LoRR, *F*[2,42] = 13.81, *p* < 0.0001; RoRR, *F*[2,40] = 9.790, *p* = 0.0003. *p < 0.05; **p < 0.01. Data are presented as Mean ± SD.

To investigate the regulatory effect of LHA^GABA^ neurons on sevoflurane general anesthesia, optogenetic AAV‐DIO‐ChR2/NpHR‐mCherry or chemogenetic AAV‐DIO‐hM3Dq/hM4Di‐mCherry viruses were microinjected into the LHA of Vgat‐Cre mice (Figure 3A,E left). After 3–4 weeks of viral expression, the efficacy of photoexcitation and chemogenetic stimulation was verified using in vitro brain slices (Figure 3D,G). The consciousness of mice under sevoflurane anesthesia was evaluated by assessing their righting reflexes during induction of and emergence from anesthesia (Figure 3A,E right). Compared with control groups, photoactivation of LHA^GABA^ neurons prolonged the LoRR time and shortened the RoRR time (Figure 3B,C). Conversely, optogenetic inhibition of LHA^GABA^ neurons prolonged the RoRR time and shortened the LoRR time (Figure 3B,C). Immunofluorescence was used to confirm the accuracy and specificity of viral expression (Figure 3F). Compared with control groups, chemogenetic activation of LHA^GABA^ neurons prolonged the LoRR time and shortened the RoRR time (Figure 3H,I). Chemogenetic Inhibition of LHA^GABA^ neurons has an opposing effect on LoRR but not a significant difference on RoRR time (Figure 3H,I). The results of both optogenetic and chemogenetic manipulations show that LHA^GABA^ neurons may regulate anesthesia‐arousal transition.

**FIGURE 4:**
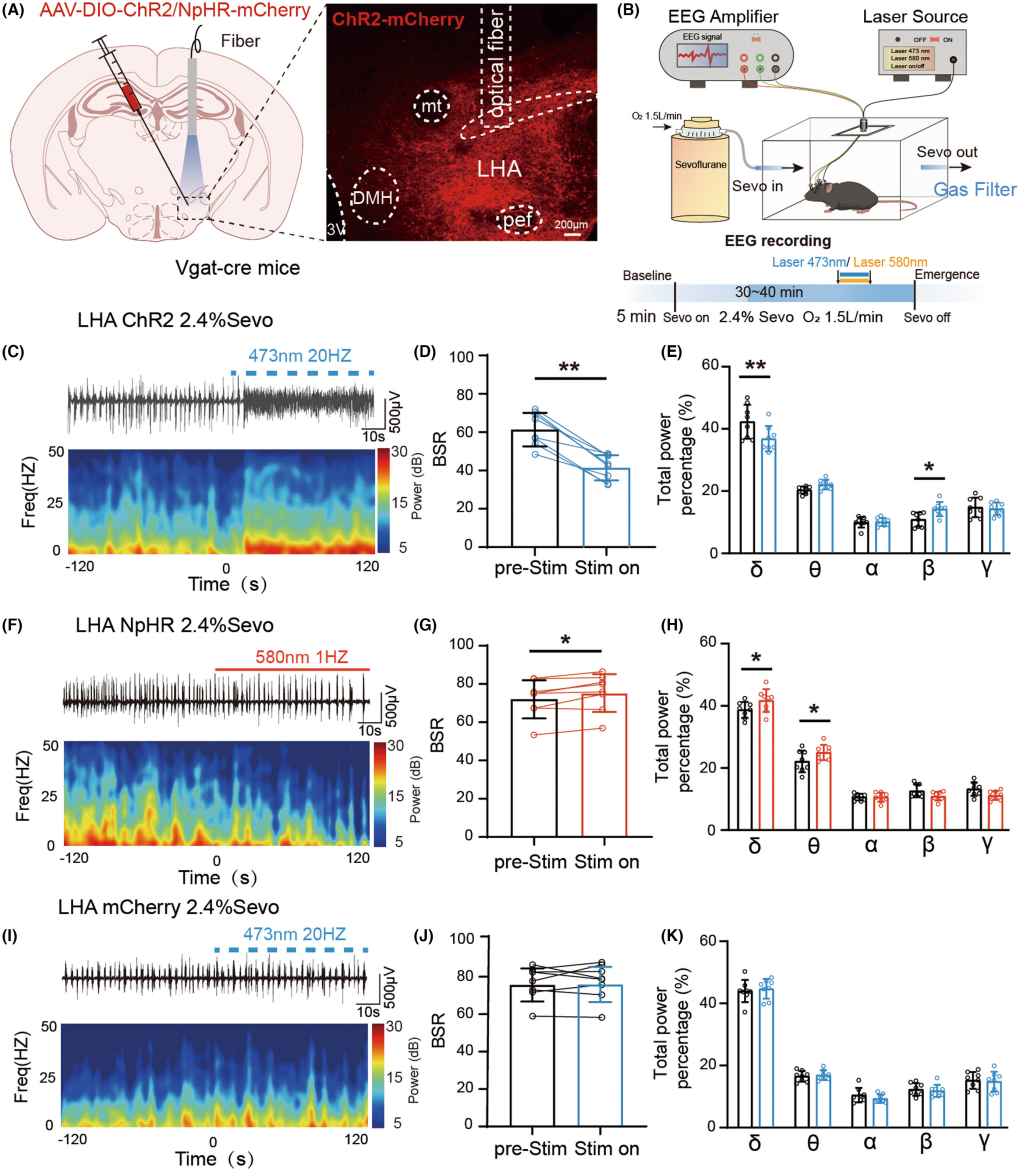
Optogenetic manipulation of LHA^GABA^ neurons alters sevoflurane anesthesia. (A) Diagrams of coronal brain sections showing the site of virus injection and optical fiber embedding (left). A typical photomicrograph showing the site of microinjection and virus transfection efficiency (right). (B) Schematic diagram of the apparatus used for behavioral observations, optical fiber stimulations, and EEG recordings during anesthesia. (C) Representative raw EEG trace (top) and the corresponding power spectrogram (bottom) during blue laser stimulation in a ChR2‐expressing mouse. (D) Compared to pre‐stimulation, activation of LHA^GABA^ neurons significantly reduces the BSR during sevoflurane maintenance. Two‐tailed paired Student's *t*‐test: *t*[7] = 7.992, *p* < 0.0001. (E) Quantification of EEG power pre and during optogenetic activation of CHR2 mice (δ: Stim on vs. pre‐stim; *p* = 0.002, β: Stim on vs. pre‐stim; *p* = 0.021). (F) Representative EEG traces during yellow laser stimulation in an NpHR‐expressing mouse. (G) Compared to pre‐stimulation, inhibition of LHA^GABA^ neurons increases the BSR during sevoflurane maintenance. Two‐tailed paired Student's *t*‐test: *t*[7] = 3.190, *p* = 0.0153. (H) Quantification of EEG power pre and during optogenetic inhabitation of NpHR mice (δ: Stim on vs. pre‐stim; *p* = 0.012, θ: Stim on vs. pre‐stim; *p* = 0.017). (I) Representative raw EEG trace (top) and the corresponding power spectrogram (bottom) during blue laser stimulation in a mCherry‐expressing mouse. (J) BSR 2 min before and 2 min after optical stimulation in mCherry‐expressing mice. Two‐tailed paired Student's *t*‐test: *t*[7] = 0.15, *p* = 0.8847. (K) Quantification of EEG power pre and during optogenetic activation of mCherry mice. There was no significant change between pre‐stim and stim on. *p < 0.05; **p < 0.01. Data are presented as Mean ± SD.

To verify the anesthesia depth change by modulation of LHA^GABA^ neurons during sevoflurane anesthesia maintenance, burst suppression patterns and the power percentage of spectrum were observed in EEGs while optogenetic manipulation was used to modulate LHA^GABA^ neural activity. Light‐sensitive channelrhodopsin‐2 (ChR2) or halorhodopsin (NpHR) was specifically expressed in LHA^GABA^ neurons (Figure 4A), and EEG was recorded (Figure 4B). To obtain a stable burst suppression ratio (BSR) during anesthesia maintenance, the sevoflurane concentration was kept at 2.4%, resulting in a stable BSR of approximately 60%~70%. We compared the BSR 2 min pre‐stim and stim‐on during administration of the optogenetic stimulation (Figure 4C,F,I). A 20 Hz train of 473 nm blue light activated LHA^GABA^ neurons and significantly reduced BSR (Figure 4D), there was also a significant change in the power of the delta and beta wave spectrograms (Figure 4E). Using yellow laser pulses to inhibit LHA^GABA^ activation, BSR increased significantly in EEG recordings during the laser‐on period compared to that before optogenetic stimulation (Figure 4G). Meanwhile, the percentage of delta and theta power in the total spectrum were increased (Figure 4H). In the control group, BSR values and power of spectrograms have no change before and after blue laser stimulation (Figure 4J,K). Overall, these EEG results show that optogenetic activation of LHA^GABA^ neurons reduced the anesthesia depth as shown on the EEG pattern.

### 3.3 Disinhibition of the LHA^GABA^‐LPAG pathway rapidly activates LPAG^Glu^ neurons

**FIGURE 5:**
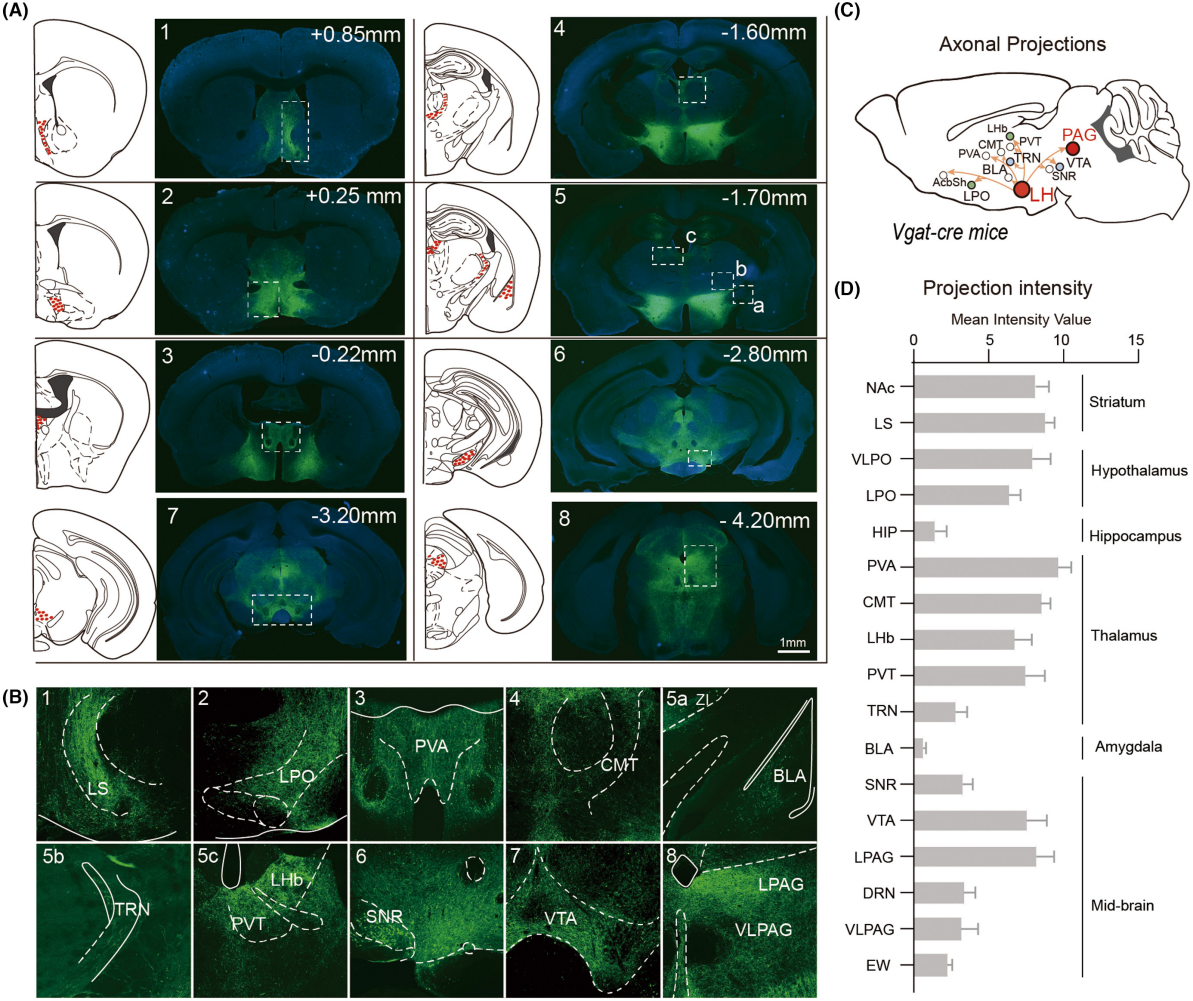
Whole‐brain mapping of axonal projections from LHA^GABA^ neurons. (A, B) Fluorescent images showing axons of neurons expressing AAV‐EF1α‐DIO‐eGFP in the AcbSh, LPO, PVA, CMT, BLA, TRN, PVT, LHb, SNR, VTA, and LPAG. (C) Main axonal projections of LHA^GABA^ neurons. (D) Distribution intensity of LHA^GABA^ axonal innervation in each nucleus. AcbSh, accumbens nucleus shell; BLA, basolateral amygdala; CMT, central medial thalamus; DRN, dorsal raphe nucleus; EW, Edinger‐Westphal nucleus; LHA, lateral hypothalamus; LHb, lateral habenular nucleus; LPAG, lateral periaqueductal gray; LPO, lateral preoptic area; PVA, periventricular; PVT, paraventricular thalamic nucleus; SNR, substantia nigra pars reticulate; TRN, thalamic reticular nucleus; VLPAG, ventrolateral periaqueductal gray; VTA, ventral tegmental area.

**FIGURE 6:**
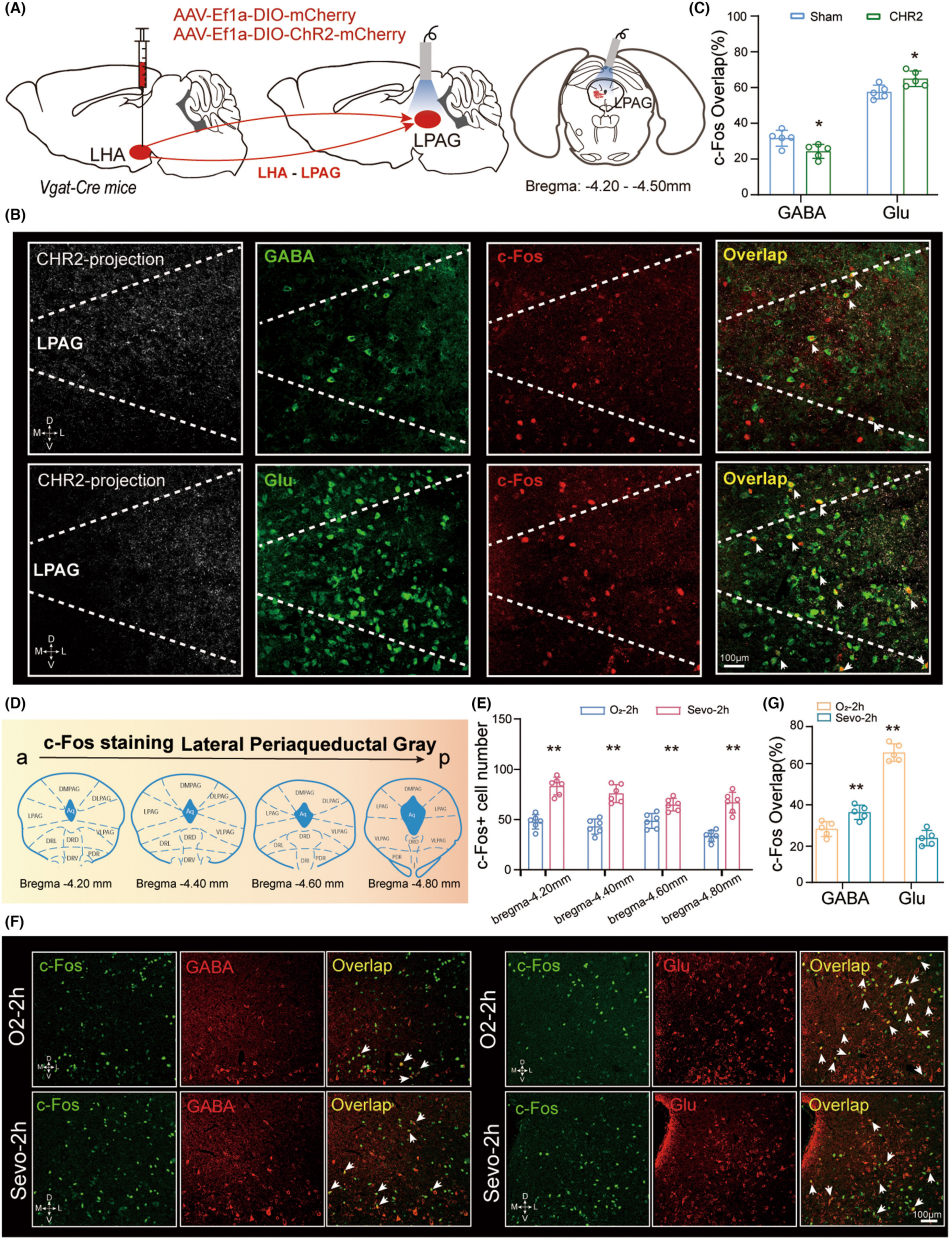
Comparison of the activity of LPAG neurons during sevoflurane anesthesia. (A) Schematic diagram of optogenetic virus injection and laser stimulation. (B) Representative immunofluorescence showing overlapping LHA^ChR2^ projection (white), GABA/Glu (green), and c‐Fos (red) stainings in the LPAG after 473 nm optical laser stimulation for 20 min. (C) c‐Fos activity of LPAG^GABA^ and LPAG^Glu^ neurons. LPAG^Glu^ neurons are activated (95% confidence interval, −13.58 to −0.894) following optogenetic stimulation, whereas LPAG^GABA^ neurons are inhibited (95% confidence interval, 0.9133 to −13.60). (D) Schematic illustration of the brain slice experiments after inhalation of sevoflurane anesthesia or pure oxygen. (E) LPAG neural activity increases from rostral to caudal. Two‐way ANOVA followed by post hoc Bonferroni's multiple comparisons: *F*[3,40] = 3.866, *p* = 0.0003. There are eight slices from 4 mice in each group. (F) Representative micrographs showing overlapping GABA/Glu (red) and c‐Fos (green) stainings in the LPAG after 2 h of oxygen exposure (O_2_‐2h) or sevoflurane anesthesia (Sevo‐2h). (G) c‐Fos activity in LPAG GABAergic and glutamatergic neurons was compared between oxygen exposure (O_2_‐2h) and sevoflurane anesthesia (Sevo‐2h). Two‐way ANOVA followed by post hoc Bonferroni's multiple comparisons: *F*[1,16] = 51.06, *p* < 0.0001. There are eight slices from four mice in each group. The results indicate that GABAergic neurons in the LPAG were activated, whereas glutamatergic neuronal activity was inhibited by sevoflurane anesthesia. *p < 0.05; **p < 0.01. Data are presented as Mean ± SD.

We used anterograde tracing to characterize and map the neurons innervated by LHA^GABA^ neurons in whole‐brain slices. Firstly, we injected AAV‐Ef1a‐DIO‐mCherry into the LHA of Vgat‐Cre mice. LHA^GABA^ neurons are projected to multiple areas of the brain (Figure 5A,B). Nucleus accumbens, LPO, PVT, VTA, and lateral periaqueductal gray (LPAG) nuclei showed high neural projections (Figure 5C,D). We found virus‐labeled neurons in various brain regions implicated in arousal, including the VTA and TRN. Unexpectedly, we also observed projections from LHA^GABA^ neurons to brain regions implicated in motivation and pain responses, such as the basolateral amygdala and LPAG. These brain areas have also been implicated in modulating stress and depression, suggesting the role of LHA^GABA^ neurons in this regulation. Since the projection of LHA^GABA^‐LPAG is one of the significantly higher areas in the midbrain, which is a crucial brain area of sleep, emotion, and anesthesia regulation, we focus on this projection for the next step of research. To explore the functional connections between LHA^GABA^ projections and LPAG neurons, we optogenetically activated selectively LPAG neurons downstream of LHA^GABA^ projections (Figure 6A). To identify the excitatory or inhibitory effects of LHA^GABA^ projections on LPAG^GABA^ or LPAG^Glu^ neurons, we immunohistochemically characterized their projections using anti‐GABA or anti‐glutamate antibodies with overlapping changes in c‐Fos expression after optogenetic stimulation of projection terminal Of LHA^GABA^ to LPAG (Figure 6B). c‐Fos‐positive neurons were mainly localized to glutamatergic, not GABAergic neurons in the LPAG, which indicates that activation of LHA^GABA^ projection induces LPAG^Glu^ excitation (Figure 6C). Further tests were performed on the LHA^GABA^‐LPAG projection neuron types. Vgat‐iCre: RosaTD (Ai 26) mice with specific encoding mCherry on GABAergic neurons were used in this experiment. We microinjected AAV‐DIO‐GCaMP6f virus into the LHA of Vgat‐iCre: RosaTD (Ai 26) mice and allowed 3 weeks for gene expression (Figure S3A). The virus was specifically expressed on LHA^GABA^ neurons and projected several fibers downstream of LPAG (Figure S3B). The GABAergic neurons in the LPAG were labeled with mCherry (red), LHA^GABA^ axon terminals were presented in GFP (green), while LPAG glutamatergic neurons were stained in pink (Figure S3C). A high proportion of GFP‐positive dots were observed surrounding LPAG^GABA^ neurons, suggesting that the axons of LHA^GABA^ neurons are in touch with the soma of GABAergic neurons in the LPAG. These results show that LHA^GABA^ neurons exerted a disinhibitory effect on LPAG^Glu^ neurons. To verify the activity of LPAG neurons during sevoflurane anesthesia, the c‐Fos expression of LPAG^Glu^ and LPAG^GABA^ neurons were examined (Figure 6D, Figure S3D). c‐Fos expression increased from rostral to caudal after 2 h of sevoflurane anesthesia (Figure 6E). Compared to the oxygen inhalation group, the percentage of c‐Fos‐positive LPAG^GABA^ neuron was significantly increased, whereas that of LPAG^Glu^ neuron was decreased in the sevoflurane anesthesia group (Figure 6F,G). These results indicate that LPAG^Glu^ and LPAG^GABA^ neurons play opposing roles in the induction of sevoflurane anesthesia.

### 3.4 LHA^GABA^ projections to the LPAG promote emergence during sevoflurane anesthesia

**FIGURE 7:**
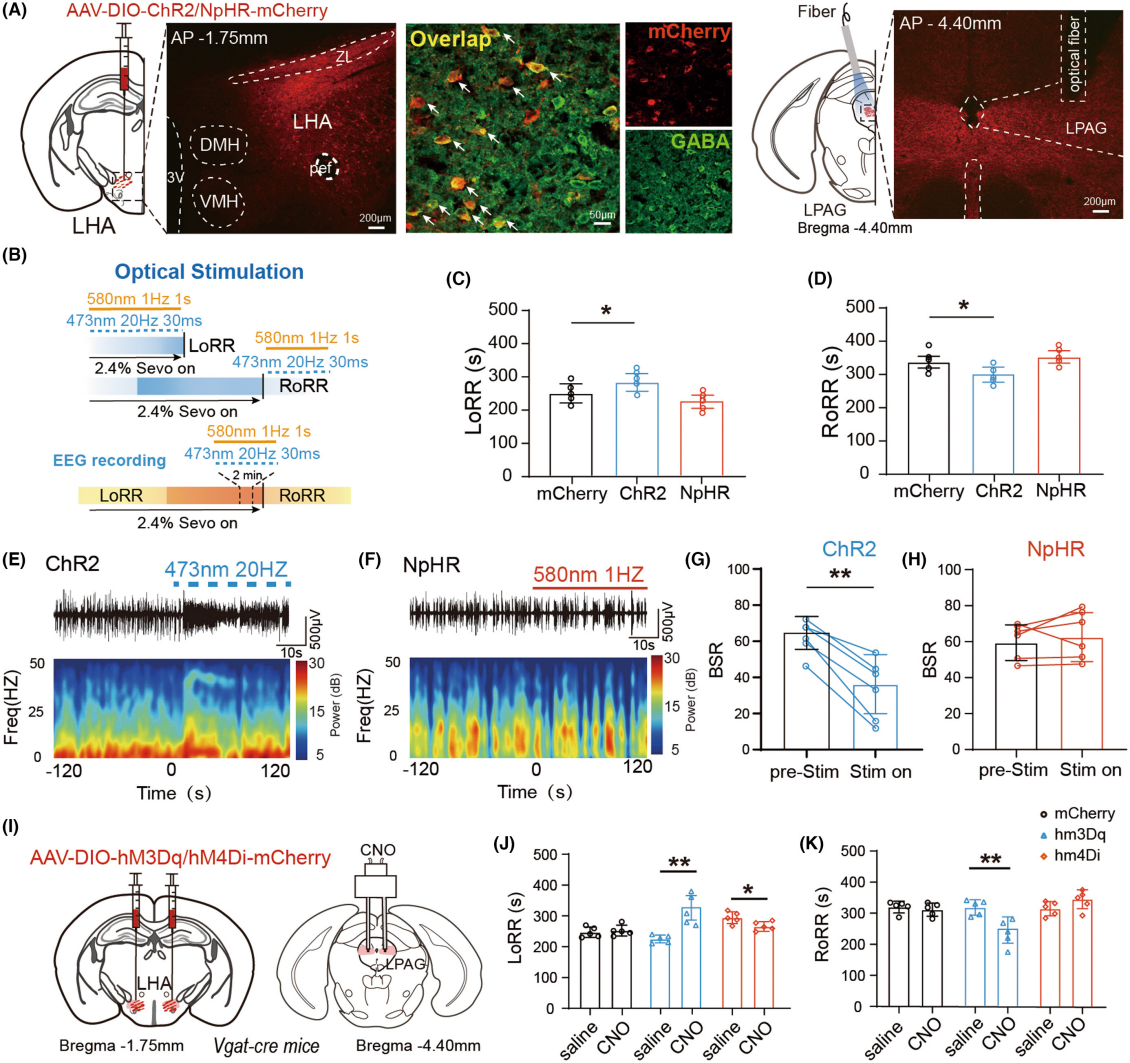
The LHA^GABA^‐LPAG circuit significantly affects arousal under sevoflurane anesthesia in mice. (A) Diagram showing the injection of optogenetic viruses into the LHA and optical fiber insertion into the LPAG of Vgat‐Cre mice. Fluorescent images show virus expression, fiber position, and overlap of LHA^GABA^ neurons. (B) Time course of the experiment. (C, D) Activation or inhibition of LHA^GABA^‐LPAG projections in response to anesthesia induction and emergence. One‐way ANOVA followed by post hoc Bonferroni's multiple comparisons: LoRR, *F*[2,27] = 0.6234, *p* = 0.037; RoRR, *F*[2,27] = 4.325, *p* = 0.024. (E, F) Representative raw EEG trace (top) and corresponding power spectrogram (bottom) during laser stimulation of optical‐expressing mice during sevoflurane anesthesia. (G) Compared to pre‐stimulation, activation of LHA^GABA^‐LPAG projections significantly reduces BSR during sevoflurane maintenance. Two‐tailed paired Student's *t*‐test: *t*[5] = 6.714, *p* = 0.0011. (H) Compared to pre‐stimulation, inhibition of LHA^GABA^‐LPAG projections has no significant effect on BSR during sevoflurane maintenance. Two‐tailed paired Student's *t*‐test: *t*[5] = 0.7973, *p* = 0.4615. (I) Illustration of the experimental equipment for DREADDs. (J, K) Effect of activation or inhibition of LHA^GABA^‐LPAG projections on the induction time and emergence time from sevoflurane anesthesia. Two‐way ANOVA followed by post hoc Bonferroni's multiple comparisons: LoRR, *F*[2,28] = 11.32, *p* = 0.0002; RoRR, *F*[2,26] = 10.44, *p* = 0.0005. *p < 0.05; **p < 0.01. Data are presented as Mean ± SD.

To explore the role of LHA^GABA^ axonal terminals in the LPAG during sevoflurane anesthesia, AAV‐DIO‐ChR2/NpHR‐mCherry or control AAV‐DIO‐mCherry virus was microinjected into the LHA of Vgat‐Cre mice, and optical fibers were embedded in the LPAG. Immunofluorescence staining confirmed the specificity of viral expression (Figure 7A). EEG was recorded during optical modulation as described above (Figure 7B). Representative EEG changes in burst suppression patterns were observed within 2 min before and after the light stimulus (Figure 7E,F). During sevoflurane maintenance, blue light pulses activated the LHA^GABA^‐LPAG projection, and BSR was significantly decreased during optogenetic stimulation (Figure 7G). However, when yellow laser pulsing was used to inhibit GABAergic terminals in the LPAG; BSR did not significantly change (Figure 7H). These results indicate that activating LHA^GABA^ terminals in the LPAG can remarkably promote arousal from anesthesia.

We also performed optical stimulation during the induction and emergence periods under sevoflurane anesthesia (Figure 7A,B). Optogenetic activation of LHA^GABA^ terminals in the LPAG prolonged LoRR time and shortened RoRR time (Figure 7C,D). Inhibition of LHA^GABA^ neurons did not affect LoRR or RoRR times (Figure 7C,D). These results indicate that optogenetic activation of the LHA^GABA^‐LPAG pathway prolongs induction and reduces emergence time under sevoflurane anesthesia.

Next, chemogenetic viruses were microinjected into the LHA, and a cannula was inserted into the LPAG of Vgat‐Cre mice (Figure 7I). Chemogenetic stimulation was combined with sevoflurane anesthesia, as described above. Compared to controls, activation of LHA^GABA^ terminals in the LPAG prolonged LoRR time and shortened RoRR time (Figure 7J,K). Inhibition of LHA^GABA^ terminals in the LPAG slightly affected LoRR but not RoRR time (Figure 7J,K).

## 4 DISCUSSION

In the present study, we aimed to test the hypothesis that LHA^GABA^ neurons play a crucial role in modulating the anesthesia‐arousal transition during sevoflurane anesthesia. LHA^GABA^ neuronal activity was significantly suppressed by sevoflurane anesthesia, as evidenced by both in vitro immunostaining and in vivo fiber photometry. Combined with chemogenetic, optogenetic, EEG, and behavioral tests, we established that the activation of LHA^GABA^ neurons reduces BSR during anesthesia maintenance and facilitates emergence from sevoflurane anesthesia. Furthermore, using anterograde tracing, we identified relevant projection areas of LHA^GABA^ neurons possibly related to arousal from anesthesia. Our findings revealed an LHA^GABA^‐LPAG pathway essential for the rapid alteration of whole‐brain states and facilitation of arousal from sevoflurane anesthesia.

In the transition of natural sleep, numerous subcortical arousal circuits have been identified to be involved in the modulation of the brain.^6^ Our previous study found that activation of VTA^GABA^ terminals in the LHA promotes NREM and REM sleep, demonstrating their role in sleep induction.^31^, ^32^ Disruption or inhibition of VTA^GABA^ neurons leads to sustained wakefulness, whereas activation of VTA^GABA^ neurons induces profound sedation.^33^ Moreover, VTA^GABA^ projections to the LHA facilitated anesthetic isoflurane effects during induction and maintenance. These projections similarly affected sleep and anesthesia, including an increased depth of unconsciousness, improved induction, and delayed recovery. However, other reports have shown that activation of preoptic GABAergic neurons modulates sleep–wake but not anesthetic state transitions.^8^, ^34^ It becomes increasingly evident that different mechanisms govern general anesthesia‐induced loss of consciousness and natural sleep.^5^, ^35^, ^36^, ^37^ Therefore, the role of various nuclei in sleep and under different anesthetics should be investigated.^12^, ^37^

The hypothalamus comprises various neuronal types with diverse molecular signatures and is a core player in regulating multiple fundamental physiological behaviors, implicating its complex role in consciousness modulation.^12^, ^24^ The LHA GABAergic neurons have traditionally been considered to be important for survival, feeding, and consciousness transition.^20^, ^23^, ^24^ The direct inhibitory effect of anesthetics on any wake‐active regions, such as the VTA, is predicted to destabilize wakefulness and promote hypnosis.^38^ In the current study, we also demonstrated that the activation of LHA^GABA^ neurons accelerated emergence and reduced the BSR during sevoflurane anesthesia. Because electrical microstimulation can activate local neuron somata as well as the pass‐by axons of neurons from other brain regions, the precise LHA GABAergic neural circuits underlying promote arousal especially general anesthetic induced conscious transition remain to be dissected precisely.^13^, ^25^ The interaction between LHA^GABA^ neurons and multiple brain regions regulating anesthesia‐arousal remains elusive, suggesting complex arousal control through interactions between long‐range connections.

Classical transection studies suggest that apart from the hypothalamus, the brainstem is essential for sustaining wakefulness.^26^ Anatomical and physiological experiments were conducted to characterize that the LHA neurons projecting to the PAG may coordinate neuronal activity in multiple brain regions, such as in feeding and conscious behavior. PAG‐projecting LH neurons also project to other brain regions such as the thalamus and lateral habenular nucleus (LHb), and along the medial forebrain bundle (MFB) to the midbrain ventral tegmental area and substantia nigra compacta (SNc), as well as to the reticular nucleus posterior to the PAG.^25^, ^39^ However, whether the LHA^GABA^ mediates the transition of consciousness induced by general anesthesia through LHA^GABA^‐PAG pathway remains unknown. Among the numerous downstream brain regions of LHA^GABA^ neurons, the brainstem PAG is functionally segmented by distinct afferent and efferent connections, molecular profiles, and associated behaviors, specifically its lateral (LPAG) and ventrolateral (VLPAG) sections are implicated in modulating various complex behaviors, including pain perception, motivation, anxiety, responses to threats, and aggression.^13^, ^25^, ^26^, ^27^ The LPAG is a heterogeneous nucleus containing mainly glutamatergic and GABAergic neurons. Earlier studies have demonstrated that LPAG^Vgat^ neurons support seeking and attacking behaviors, whereas LPAG^Vglut2^ neurons exclusively support attacking behaviors.^25^, ^40^ This implies varied regulatory functions among different types of LPAG neurons. The LPAG is also engaged in sleep–wake regulation, as LPAG neurotensinergic neurons promote NREM sleep.^28^ Our results further proved that the GABAergic LHA projection to the LPAG plays a pro‐arousal role in sevoflurane anesthesia. Activation of the LHA GABAergic projections to the LPAG reduced the BSR during sevoflurane maintenance and changed the anesthetic behaviors. Compared to neural circuits stimulation and c‐Fos staining, our results suggested that LHA^GABA^ neurons mediate the disinhibition of LPAG glutamatergic neuronal activity. This disinhibition effect of LPAG Glu neurons could have contributed to the inhibition of local GABA neurons in LPAG, thereby facilitating arousal following sevoflurane exposure in mice. Consistent with previous and our results, we hypothesized that GABA release from LHA^GABA^ neurons inhibits GABAergic neurons within the LPAG area, thereby disinhibiting LPAG^Glu^ neurons from projecting further to arousal‐associated brain regions and affecting sevoflurane anesthesia. In concert, these findings provide a mechanism for how LHA^GABA^ neurons contribute to sevoflurane‐induced unconsciousness via their projections to the LPAG, elucidating the inhibitory pathway's role in promoting arousal from general anesthesia through a classical disinhibitory effect.

However, our study also has some limitations. First, only male mice were used. Our previous study found male mice were more sensitive to sevoflurane, representing sex differences in response to general anesthesia through the estrogen receptor alpha (ERα) in the medial preoptic area.^30^ As another sexually dimorphic region, LPAG contains numerous ERα‐immunopositive neurons distributed in a species‐specific way.^41^ Therefore, further research is needed to examine whether our findings can be extrapolated to females. Second, high‐frequency stimulation in the downstream could induce antidromic activation and cause retrograde depolarization of upstream starting cell bodies and then induce an excitatory effect through other projections; we have not excluded this possibility in this study.

In conclusion, this study identified that inhibitory LHA^GABA^ neural projections to the LPAG play a pro‐emergency role in consciousness transitions during sevoflurane anesthesia. Our results added a piece of new evidence to support that the inhibitory neural pathways in specific brain regions are involved in facilitating the arousal from general anesthesia. These findings enhance our understanding of the mechanisms of consciousness shift and potentially facilitate the development of studies involving the GABAergic system. It also provides new insights into the discovery of novel neural targets for comparing the roles of central neuronal pathways in sleep and general anesthesia.

## AUTHOR CONTRIBUTIONS

Hailong Dong and Dan Wang initiated and directed the study. Hailong Dong and Huiming Li revised the manuscript. Dan Wang and Huiming Li wrote the manuscript draft. Chang Bao, Huimin Wu, Xinxin Zhang, Fang Zhou and Sa Wang performed the experiments. Dan Wang, Jiannan Li and Huimin Wu analyzed the data. All authors contributed to and approved the manuscript.

## CONFLICT OF INTEREST STATEMENT

No conflict of interest exists in the submission of this manuscript, and the manuscript is approved by all authors for submission to CNS Neuroscience & Therapeutics.

## Supporting information


Table S1

Table S2

Appendix S1

## Data Availability

The data that support the findings of this study are available from the corresponding author upon reasonable request.
