# Supplementary material for: A hypothalamus‐lateral periaqueductal gray GABAergic neural projection facilitates arousal following sevoflurane anesthesia in mice
Source: CNS Neurosci Ther. 2024 Sep 24;30(9):e70047. doi: 10.1111/cns.70047 (PMC11421888; doi:10.1111/cns.70047)
Supplement: Supplementary file 1 — Table S1 [file CNS-30-e70047-s001.docx]

**Key Resources Table**

| **Reagent or Resource** | **Source** |
| --- | --- |
| Experimental models: Organisms | |
| Mouse: C57BL/6 | The Fourth Military Medical University’s Animal Experimental Center |
| Mouse: Vgat-Cre | the Jackson ImmunoResearch Laboratory |
| Mouse: Vglut2-Cre | the Jackson ImmunoResearch Laboratory |
| Mouse: Rosa26 | the Jackson ImmunoResearch Laboratory |
| Antibodies | |
| G.P anti-c-Fos | Abcam |
| Rabbit anti-Glu | GeneTex |
| Rabbit anti-GABA | GeneTex |
| Mouse anti-OA | GeneTex |
| Rabbit anti-MCH | GeneTex |
| Anti-G.P 594 | Jackson ImmunoResearch |
| Anti-rabbit 488 | Jackson ImmunoResearch |
| Anti-mouse 488 | Jackson ImmunoResearch |
| Virus strains | |
| rAAV-Ef1α-DIO-ChR2-mCherry | Brain-VTA, Wuhan, China |
| rAAV-Ef1a-DIO-NpHR3.0-mCherry | Brain-VTA, Wuhan, China |
| rAAV-Ef1a-DIO-mCherry | Brain-VTA, Wuhan, China |
| AAV-Ef1a-DIO-hM3Dq-mCherry | Brain-VTA, Wuhan, China |
| AAV-Ef1a-DIO-hM4Di-mCherry | Brain-VTA, Wuhan, China |
| AAV-DIO- GCaMP6f | Brain-VTA, Wuhan, China |
| Software and algorithms | |
| ImageJ | National Institutes of Health, USA |
| Prism 9.0 | GraphPad Software Inc, San Diego, CA, USA |
| PowerLab 16/35 amplifier system | PL3516, AD Instruments, Dunedin, New Zealand |
| LabChart Pro version 8.1.13 software | MLU60/8, AD Instruments, Dunedin, New Zealand |
| Matlab | MathWorks |
| Instruments | |
| Stereotaxic frame | RWD, China |
| Philips G60 anesthetic monitor | Philips Goldway, Germany |
| Fluorescence photometer | Thinker Tech, Nanjing, China |
| Freezing microtome | CM1900, Leica |
| Laser confocal fluorescence microscope | FV1200, Olympus |
| Optogenetic stimulator | Thinker Tech, Nanjing, China |
| Other | |
| Sevoflurane | Baxter Healthcare |
| CNO | Cayman Chemical, USA |
| Optical fiber | Inper, Hangzhou, China |

**CONTACT FOR REAGENT AND RESOURCE SHARING**

Further information and requests for resources and reagents should be directed to and will be fulfilled by the Lead Contact Prof. Hai-long Dong ( [*hldong6@hotmail.com*](mailto:hldong6@hotmail.com) ).
