# Supplementary material for: A hypothalamus‐lateral periaqueductal gray GABAergic neural projection facilitates arousal following sevoflurane anesthesia in mice
Source: CNS Neurosci Ther. 2024 Sep 24;30(9):e70047. doi: 10.1111/cns.70047 (PMC11421888; doi:10.1111/cns.70047)
Supplement: Supplementary file 3 — Appendix S1 [file CNS-30-e70047-s002.docx]

**Supplemental Materials and Methods**

**Stereotaxic Surgery**

We fixed Vgat-Cre mice or Vglut2-Cre mice to the induction chamber within 1.4-1.5 vol% isoflurane (Baxter Healthcare, Puerto Rico) vaporized by oxygen of 1.0L/min, then transferred and placed on a stereotaxic frame (RWD, Shenzhen, China) while keeping anaesthetized by 0.8% isoflurane through a mask [1,2]. The mouse scalp was then sectioned sagittally exposing a clear skull and a heating mat was used to keep the mice warm [3,4]. Mice were randomly assigned to experimental or control groups in the same home cage.

For optogenetic stimulation, 200nL rAAV-Ef1α-DIO-ChR2-mCherry (ChR2) or control virus rAAV-Ef1a-DIO-mCherry (mCherry) were microinjected into the LHA (AP: -1.75 mm; ML: -0.90 mm; DV: -5.10 mm) of Vgat-Cre mice. Subsequently, an optical fibre (2.5 mm in diameter, 200 μm fiber optical cable, NA = 0.37) was inserted into the ipsilateral LHA or the LPAG (AP: -4.40 mm; ML: -0.40 mm; DV: -2.00 mm). Optical viruses rAAV-Ef1a-DIO-NpHR-mCherry (NpHR) and optogenetic fibres were implanted bilaterally for optogenetic inhibition. To verify the regulatory role of LPAG neurons during sevoflurane anaestheisa, 200nL rAAV-Ef1α-DIO-ChR2-mCherry (ChR2) or control virus rAAV-Ef1a-DIO-mCherry (mCherry) was microinjected into the LPAG (AP: -4.40 mm; ML: -0.40 mm; DV: -2.00 mm) of Vgat-Cre mice or Vglut2-Cre mice. Subsequently, an optical fibre (2.5 mm diameter, 200 μm fibre optical cable, NA = 0.37) was inserted into the ipsilateral LPAG (AP: -4.40 mm; ML: -0.40 mm; DV: -2.00 mm). Three stainless steel screws were fastened into the subject's skull as electrodes for EEG recording [4,5]: the positive electrode on left side of the rostral (AP: +2.0 mm, ML: +1.5 mm), the negative electrode on the right side (AP: -2.0 mm, ML: -1.5 mm), and the reference electrode on the causal of the head (AP: -5.5 mm, ML: +1.5 mm), and then secured to the skull by dental cement. rAAV-Ef1a-DIO-hM3Dq-mCherry (hM3Dq), AAV-Ef1a-DIO-hM4Di-mCherry (hM4Di), or rAAV-Ef1a-DIO-mCherry (control) was bilaterally microinjected into the LHA of Vgat-Cre mice at a rate of 30 nL/min for chemogenetic investigations. For LHA terminal projection chemogenetic experiments, chemogenetic viruses were microinjected into the LHA of Vgat-Cre mice, and a cannula was inserted into the LPAG. The micropipette was carefully recovered after being left for 10 min following injection. We then used dental cement to fasten all the screws and fibers to the skull. An EEG recording and behavioral test were performed to assess the animal's healing and viral expression at least three weeks after surgery.

**Fiber Photometry Recording**

For calcium signal recording experiments, mice were microinjected with rAAV-Ef1a-DIO-GCaMP6f-WPRE into the LHA of Vgat-Cre mice. Three weeks following viral expression, an optical fibre was implanted in the LHA for photometry recording [2,3]. A beam of 488 nm successive LED light was emitted and reflected by a dichromic mirror, and the fluorescence of GCaMP6f was focused and recorded by the photometer. To reduce the bleaching of the calcium indicator, laser intensity at the fibre tip was kept between 30 and 40 µW. Calcium signal analysis was carried out on MATLAB using custom code. The ΔF/F, calculated as (F-F_0_)/F_0_, where F represents the real-time fluorescence signal, and F_0_ represents the baseline of the fluorescence signal.

**Immunofluorescence Staining**

Following the experiments, the mice were immediately sacrificed with 10% potassium chloride (KCl), transcranial perfusion with 4% paraformaldehyde (PFA) and 0.9% saline [1,3,5]. The brains were coronally sliced at a 40-m thickness. The brain slices were then cleaned in PBS before being blocked for 2 h at 24℃ with standard donkey serum at 5% in PBS containing 0.3% Triton X-100. Primary antibodies were incubated for at least 24 h at 4°C. Afterwards, the sections were washed, and the secondary antibodies were incubated for 2 h at 24℃. Finally, the brain slices were mounted and imaged using a laser confocal fluorescence microscope and ImageJ to count the number of neurons.

**Slice Recording**

We conducted a whole cell patch clamp to assess the electrophysiological characteristics in response to the activation of LHA^GABA^ neurons as previously described [1,3-5]. Mice were anaesthetized with isoflurane and decapitated; the brain was immediately removed and immersed in an ice-cold cutting medium. Coronal LHA brain slices (200-300 μm thick) were obtained in an ice-cold cutting solution bubbled with 95% O_2_ and 5% CO_2_ using a VT1200S vibratome (Leica Microsystems). Whole-cell recordings were performed using a horizontal puller (P-97, Sutter Instruments) with resistances between 4-6 MΩ and micropipettes made from borosilicate glass capillaries (1.5 mm OD, 1.1 mm ID).

**Calculation of the sample size**

We calculated the sample size in this current study through a Calculator from <https://powerandsamplesize.com/Calculators/>.

For calculating the sample size of estimation of induction and emergence time, according to our previous results [3,4], the Group 'A' mean, μA was 292.8, Group 'B' mean, μB was 331.2, Group 'A' Standard Deviation, σA was 16.1, Group 'B' Standard Deviation, σB was 28.0, Sampling Ratio, κ=nA/nB was 1. When α=0.05, β=0.2 and two-tailed analysis were involved, the sample size was calculated to be 5 mice per group via the formulas:


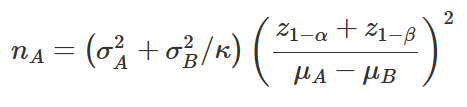


And 4-7 animals in each group were normally used in previous studies [2,3,6,7]. Thus, we used 8 mice in each group for LHA^GABA^ local stimulation and 5 mice in each group for LHA^GABA^-LPAG projection stimulation in LoRR/RoRR experiments.

For calculating the sample size of EEG (%BSR) recording, according to our previous reports [3], the Group 'A' mean, μA was 40.5, Group 'B' mean, μB was 22.4, Group 'A' Standard Deviation, σA was 2.7, Group 'B' Standard Deviation, σB was 11.8, Sampling Ratio, κ=nA/nB was 1. When α=0.05, β=0.2 and two-tailed analysis were involved, the sample size was calculated to be 3 mice. And 4-8 animals in each group were normally used in previous studies [4,8,9]. Thus, we used 8 mice in each group or no less than 6 mice in each group in EEG experiments.

For calculating the sample size of fiber photometry experiments, according to our previous reports [2,3], the Group 'A' mean, μA was 2.10, Group 'B' mean, μB was 8.28, Group 'A' Standard Deviation, σA was 0.9945, Group 'B' Standard Deviation, σB was 3.743, Sampling Ratio, κ=nA/nB was 1. When α=0.05, β=0.2 and two-tailed analysis were involved, the sample size was calculated to be 3 mice. And 4-6 animals in each group were normally used in previous studies [2,3]. Thus, we used 6 mice in each group in fiber photometry experiments.

As to anterograde tracing experiments, 3-5 mice were normally used in previous studies [10,11], so we used 5 mice to identify the downstream of LHA^GABA^ neurons and analyzed intensity values across all samples.

The success of the surgery was judged by the position of the optical fiber and the expression of the virus in each mouse, we removed the mice from results that failed virus expression or fiber placement. In the fiber photometry recording of LHA^GABA^ neurons experiment, there was one mouse died during surgery. No animal was removed from the two modulation groups in the local LHA^GABA^ cell bodies assessment. In the optical stimulation of GABAergic terminals in the LPAG experiment, there was one mouse died during surgery, we also removed one mouse from Group ChR2-mCherry (incorrect optical fiber placement) and one animal from Group mCherry (poor virus expression). In the Chemogenentic stimulation of GABAergic terminals in the LPAG experiment, there were two mice died during surgery.

**References**

[1] Zhang Y, Li H, Zhang X, et al., Estrogen Receptor-A in Medial Preoptic Area Contributes to Sex Difference of Mice in Response to Sevoflurane Anesthesia[J]. *Neurosci Bull*, 2022, 38(7):703-719. DOI:10.1007/s12264-022-00825-w

[2] Li A, Li R, Ouyang P, et al., Dorsal raphe serotonergic neurons promote arousal from isoflurane anesthesia[J]. *CNS Neurosci Ther*, 2021, 27(8):941-950. DOI:10.1111/cns.13656

[3] Zhou F, Wang D, Li H, et al., Orexinergic innervations at GABAergic neurons of the lateral habenula mediates the anesthetic potency of sevoflurane[J]. *CNS Neurosci Ther*, 2023, 29(5):1332-1344. DOI:10.1111/cns.14106

[4] Zhao S, Li R, Li H, et al., Lateral Hypothalamic Area Glutamatergic Neurons and Their Projections to the Lateral Habenula Modulate the Anesthetic Potency of Isoflurane in Mice[J]. *Neurosci Bull*, 2021, 37(7):934-946. DOI:10.1007/s12264-021-00674-z

[5] Wang D, Guo Y, Li H, et al., Selective optogenetic activation of orexinergic terminals in the basal forebrain and locus coeruleus promotes emergence from isoflurane anaesthesia in rats[J]. *Br J Anaesth*, 2021, 126(1):279-292. DOI:10.1016/j.bja.2020.09.037

[6] Solt K, Van Dort C J, Chemali J J, et al., Electrical stimulation of the ventral tegmental area induces reanimation from general anesthesia[J]. *Anesthesiology*, 2014, 121(2):311-319. DOI:10.1097/ALN.0000000000000117

[7] Yi T, Wang N, Huang J, et al., A Sleep-Specific Midbrain Target for Sevoflurane Anesthesia[J]. *Adv Sci (Weinh)*, 2023, 10(15):e2300189. DOI:10.1002/advs.202300189

[8] Li J, Li H, Wang D, et al., Orexin activated emergence from isoflurane anaesthesia involves excitation of ventral tegmental area dopaminergic neurones in rats[J]. *Br J Anaesth*, 2019, 123(4):497-505. DOI:10.1016/j.bja.2019.07.005

[9] Xu Z, Hu S W, Zhou Y, et al., Corticotropin-releasing factor neurones in the paraventricular nucleus of the hypothalamus modulate isoflurane anaesthesia and its responses to acute stress in mice[J]. *Br J Anaesth*, 2023, 130(4):446-458. DOI:10.1016/j.bja.2022.12.020

[10] Zhang Y, Zhao S, Rodriguez E, et al., Identifying local and descending inputs for primary sensory neurons[J]. *J Clin Invest*, 2015, 125(10):3782-3794. DOI:10.1172/JCI81156

[11] Hua T, Chen B, Lu D, et al., General anesthetics activate a potent central pain-suppression circuit in the amygdala[J]. *Nat Neurosci*, 2020, 23(7):854-868. DOI:10.1038/s41593-020-0632-8

**Supplemental Figures and Legends**

**Supplemental Figure 1**


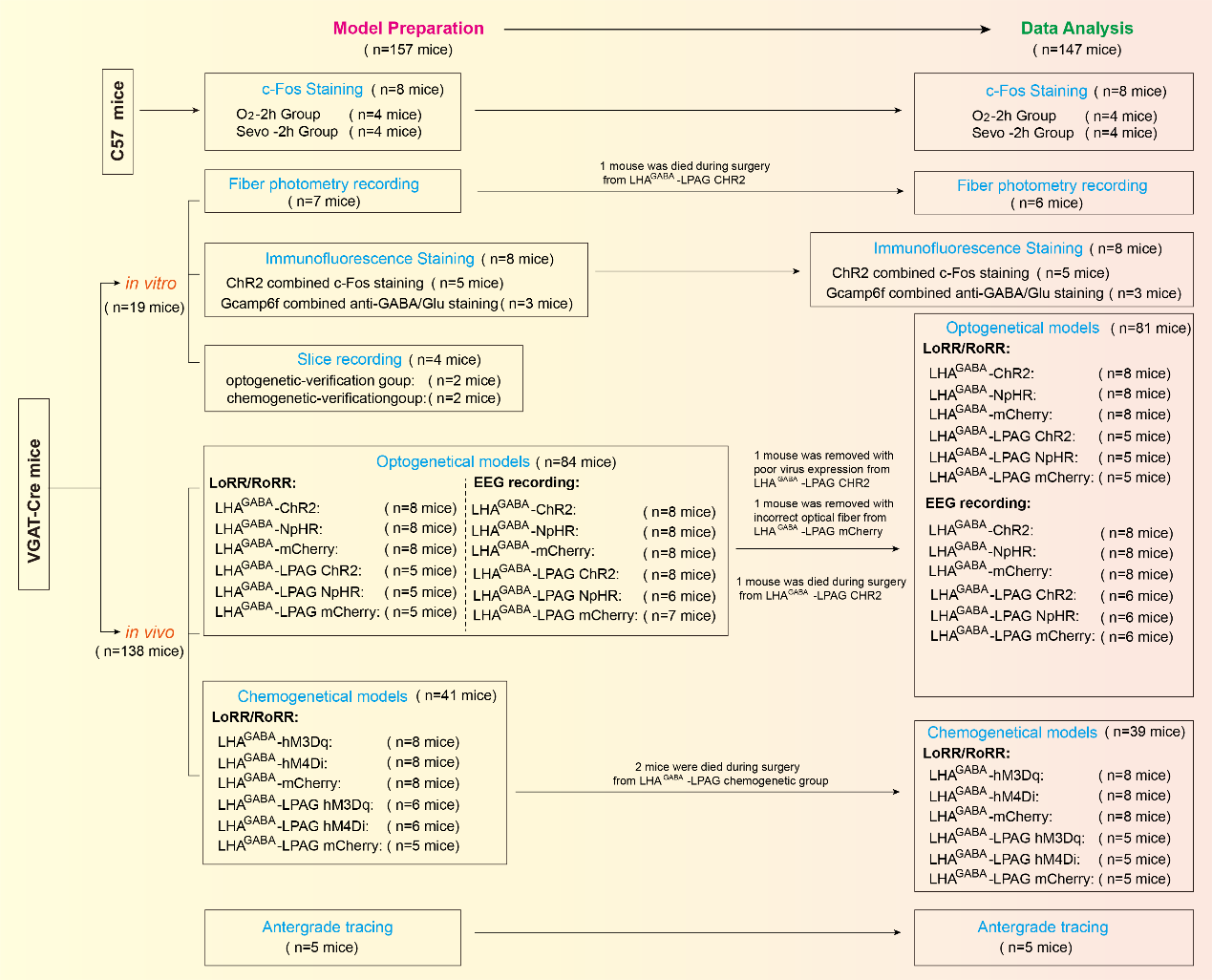


**Fig. S1 Flow Chart.** EEG indicates electroencephalogram; LHA^GABA^ -ChR2: optical excitation; LHA^GABA^ -mCherry, control virus; LHA^GABA^ -NPHR, optical inhibition; LORR, loss of righting reflex; RORR, recovery of righting reflex; LHA^GABA^ -hM3Dq: chemogenentical excitation; LHA^GABA^ -mCherry, control virus; LHA^GABA^ -hM4Di, chemogenetical inhibition; GABA, GABAergic neurons; Glu, glutamatergic neurons; LHA, lateral hypothalamus area; LPAG, lateral periaqueductal grey.

**Supplemental Figure 2**


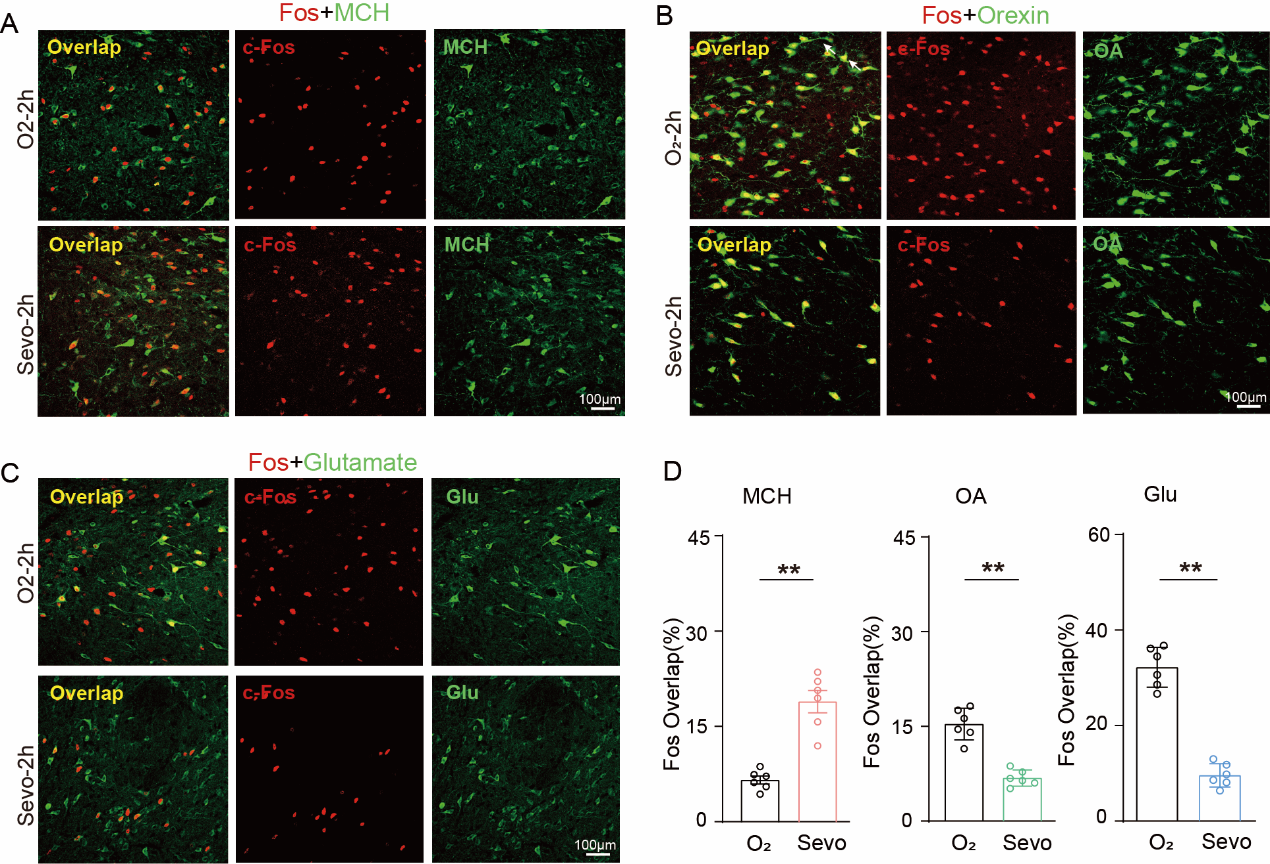


**Fig. S2 (related to Fig. 1) The activity of LHA neurones responds to sevoflurane anaesthesia.**

(A-C) Representative micrographs showing overlapping MCH/orexin/Glu (green) and c-Fos (red) staining in the LHA following 2 h of oxygen exposure (O_2_-2h) or sevoflurane anaesthesia (Sevo-2h). (D) c-Fos activity of LHA^MCH^, orexinergic, and glutamatergic neurones was compared between oxygen exposure (O_2_-2h) and sevoflurane anaesthesia (Sevo-2h). LHA^MCH^ neurones are activated by sevoflurane anaesthesia, whereas glutamatergic and orexinergic neuronal activity is inhibited. *P<0.05; **P<0.01. Data are presented as mean±SD.

**Supplemental Figure 3**


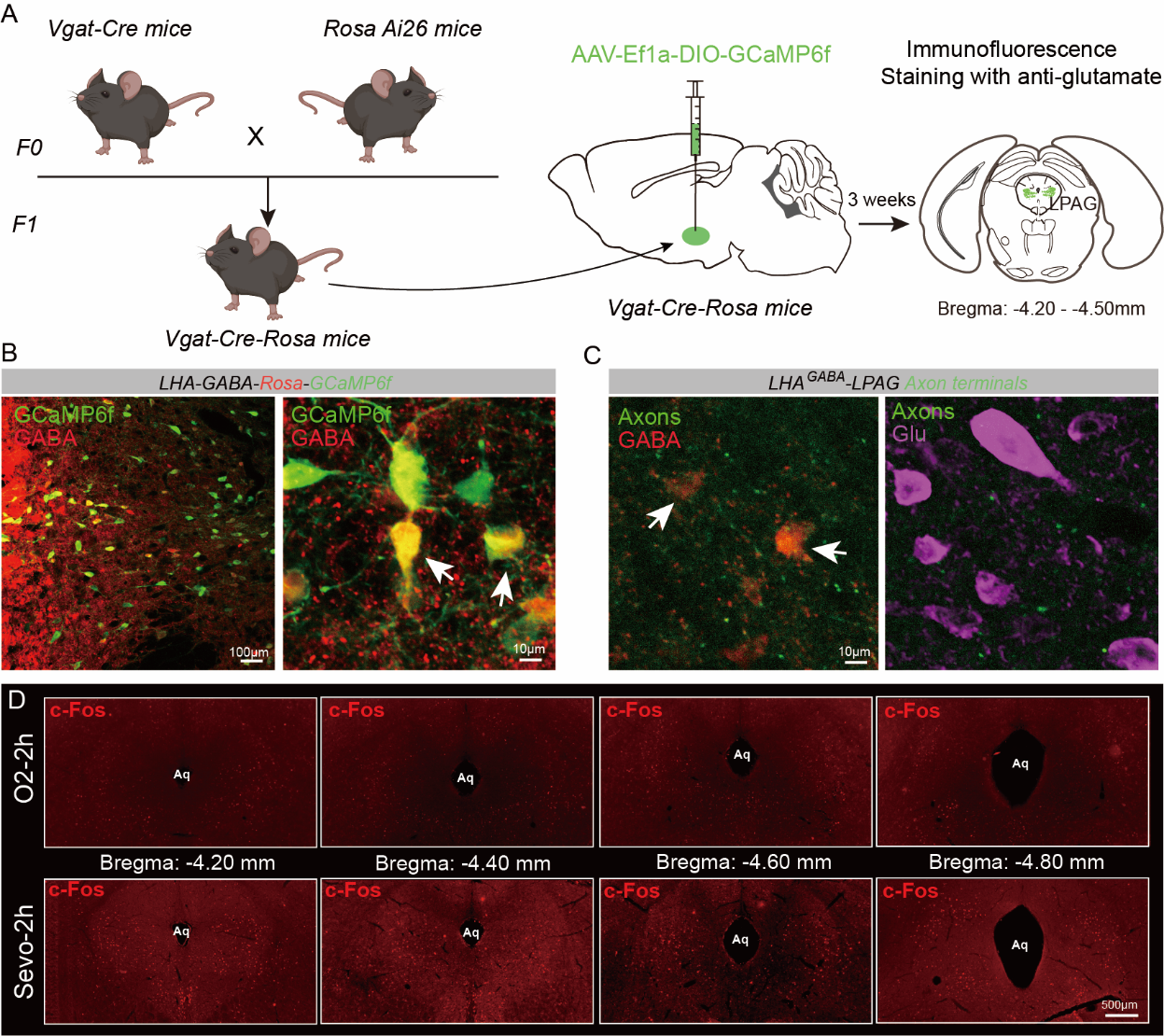


**Fig. S3 (related to Fig.5) Activation of LHA^GABA^-LPAG circuits causes LPAG^Glu^ neuronal activity.**

(A) Experimental design of the Vgat-Cre-Rosa mice strategy (left) and diagram of GCaMP6f virus injection and immunofluorescence slice sites (right). (B) Expression of virus (GFP) in GABAergic neurones (mCherry) in the LHA. White arrows indicate GABAergic neurones expressing with the AAV-virus (right). (C) The LHA GABAergic neurones projections (Axons) to GABAergic neurones (mCherry, left) or axons overlapped with glutamatergic neurones (right) in the LPAG. White arrows indicate GABAergic terminals around the cell bodies of neurones in the LPAG. (D) Representative images of c-Fos expression in brain regions, especially near the PAG, in control and sevoflurane groups. Data are presented as Mean±SD.
